# A nanoforest-based humidity sensor for respiration monitoring

**DOI:** 10.1038/s41378-022-00372-4

**Published:** 2022-04-21

**Authors:** Guidong Chen, Ruofei Guan, Meng Shi, Xin Dai, Hongbo Li, Na Zhou, Dapeng Chen, Haiyang Mao

**Affiliations:** 1 Institute of Microelectronics of Chinese Academy of Sciences, Beijing, China; 2 University of Chinese Academy of Sciences, Beijing, China; 3 Jiangsu Hinovaic Technologies Co., Ltd, Wuxi, China

**Keywords:** Organic-inorganic nanostructures, Nanosensors, Sensors, Electrical and electronic engineering

## Abstract

Traditional humidity sensors for respiration monitoring applications have faced technical challenges, including low sensitivity, long recovery times, high parasitic capacitance and uncalibrated temperature drift. To overcome these problems, we present a triple-layer humidity sensor that comprises a nanoforest-based sensing capacitor, a thermistor, a microheater and a reference capacitor. When compared with traditional polyimide-based humidity sensors, this novel device has a sensitivity that is improved significantly by 8 times within a relative humidity range of 40–90%. Additionally, the integration of the microheater into the sensor can help to reduce its recovery time to 5 s. The use of the reference capacitor helps to eliminate parasitic capacitance, and the thermistor helps the sensor obtain a higher accuracy. These unique design aspects cause the sensor to have an excellent humidity sensing performance in respiration monitoring applications. Furthermore, through the adoption of machine learning algorithms, the sensor can distinguish different respiration states with an accuracy of 94%. Therefore, this humidity sensor design is expected to be used widely in both consumer electronics and intelligent medical instrument applications.

## Introduction

Respiration, which is the exchange of gases between a body and its surrounding environment, is a critical process for supporting human life and activity^1,2^. Abnormal respiration is usually a sign of an individual’s physical problems. Many illnesses and health conditions, including heart disease, pneumonia, bronchitis, sleep apnea and hyperpyrexia caused by infection, will cause changes in a person’s respiratory frequency and depth^3^. Humidity sensing represents a promising approach to establish a relationship between human respiration and electrical signals. Therefore, the demand for humidity sensors to be used in respiration monitoring is growing at a tremendous rate^4,5^. However, applications of conventional humidity sensors are generally limited by their large size, low sensitivity, nonnegligible temperature drift and slow responses^6,7^. Following the development of microelectromechanical systems (MEMSs) in recent decades, miniaturized humidity sensors designed using several different principles, including capacitive, resistive, resonant and optical sensors, have been developed^8–13^. The resistive devices are small, simple and low-cost, but this device type has the disadvantage of low test accuracy because of its poor antijamming capability^14^. Optical sensor devices have high test accuracy, but their micromachining processes do not match well with integrated circuit techniques^15,16^. Among the different design principles, the capacitive-type device is favored because this device offers both better accuracy over a wide relative humidity (RH) range and integration convenience^17^.

A typical capacitive humidity sensor usually consists of interdigital electrodes (IDEs) that are covered with a layer of humidity-sensitive material^18^. The performance of capacitive humidity sensors is restricted in terms of the following aspects. First, the relative scarcity of high-quality humidity-sensitive materials and the parasitic capacitance from the substrate limit both the sensitivity and the accuracy of these sensors. To date, the sensitive materials used in these devices mainly include porous silicon, ceramics and organic materials^19–25^. Organic materials such as polyimide (PI) have been widely used. However, PI is a porous material that contains semienclosed nanostructures^26^, and the full output range of PI-based humidity sensors is usually only 1 to 2 pF, which means that high-precision and high-cost application-specific integrated circuits (ASICs) are required to match these sensors^27,28^. Porous silicon and ceramics, such as anodic aluminum oxide, exhibit better sensitivity because they offer better hygroscopicity, but the preparation processes for these materials are relatively complex and are not compatible with conventional complementary metal-oxide-semiconductor (CMOS) processing^29^. In addition, Gerwen’s research^30^ indicates that parasitic capacitance appears when the individual electric field lines pass through the substrate, which will then affect both the true value test for the humidity-sensitive capacitance and the accuracy of the sensors^31^. In addition, in practical applications, the ambient temperature affects the dielectric constant of humidity-sensitive materials, which causes the capacitance to change and thus causes inaccurate measurement results^32,33^. Furthermore, when humidity sensors of this type are used in high RH environments for long periods, the condensation of water vapor usually occurs on the sensor surface. This water condensation process means that desorption of the water molecules takes a relatively longer time and thus leads to longer recovery times for these sensors^34,35^. To address these challenging issues, a humidity-sensitive device configuration that can not only eliminate parasitic capacitance and calibrate the temperature drift but can also increase sensitivity and reduce the recovery time of the humidity sensors is strongly needed.

In this work, a novel triple-layer humidity sensor comprising a sensing capacitor, a reference capacitor, a microheater and a thermistor is reported. In this sensor, the sensing capacitor uses in situ integrated nanoforests as the humidity-sensitive material. Because these nanoforests are hygroscopic and are composed of fully open nanostructures, this humidity sensor exhibits high sensitivity. In addition, a reference capacitor is integrated into the structure to prevent environmental jamming and parasitic capacitance generation, a thermistor is added to provide temperature compensation, and a microheater is used to further accelerate the desorption of water molecules. With all these specific design features, the proposed sensor demonstrates excellent sensitivity, fast recovery speeds, high accuracy and improved antijamming capability. Furthermore, the humidity sensor was implanted into an N95 mask for monitoring different respiratory states, and an accuracy for monitoring respiratory states ranging up to 94% was achieved through the adoption of machine learning algorithms. The proposed sensor is expected to have wide-ranging applications in consumer electronics and medical instruments.

## Results and discussion

### Design and fabrication

**Fig. 1:**
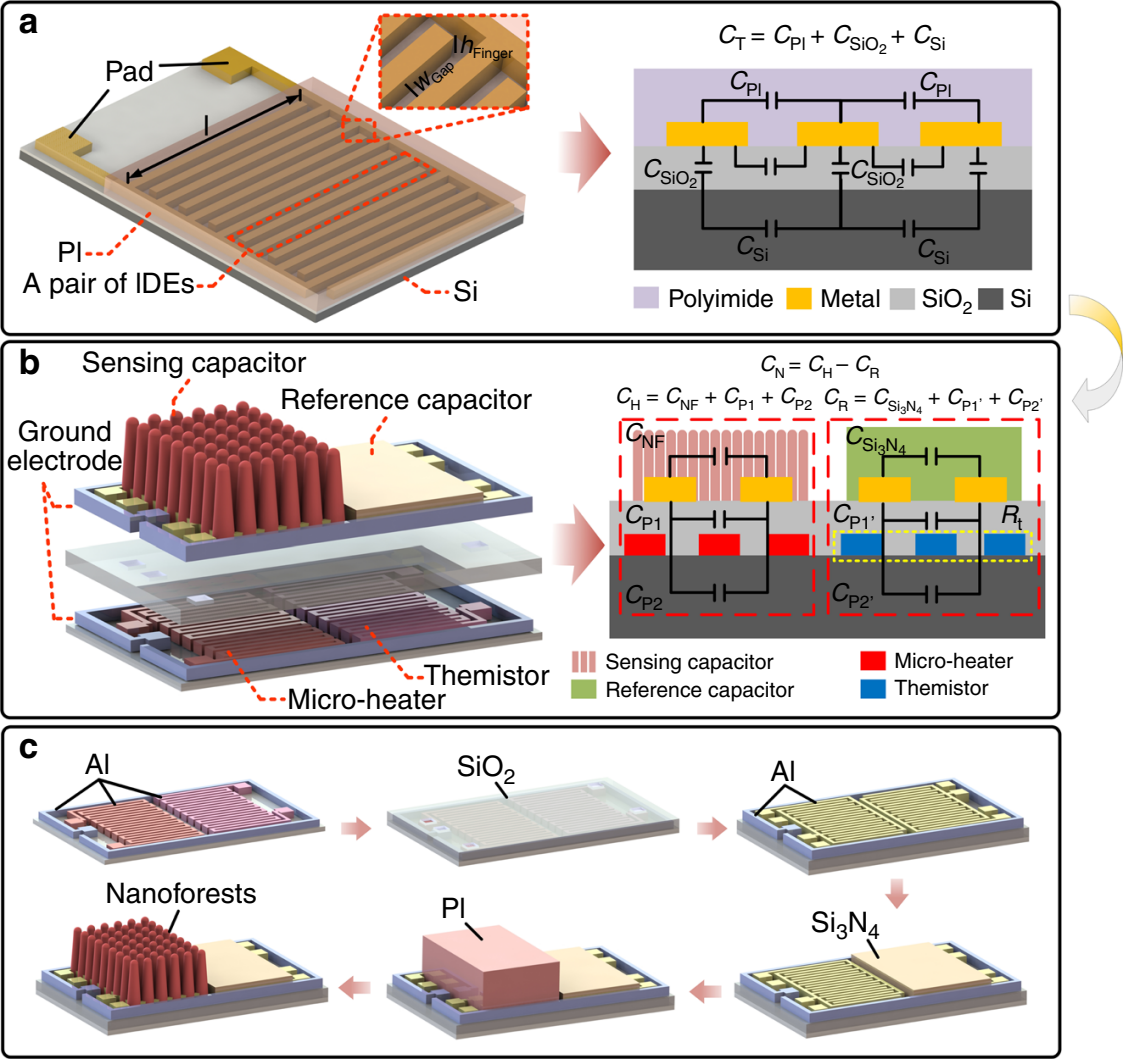
Comparison of the structure and fabrication process between the traditional capacitive humidity sensor and the novel humidity sensor. **a** Schematic diagram of structure and capacitance distribution of a traditional capacitive humidity sensor. **b** Schematic diagram of structure and capacitance distribution of the proposed triple-layer humidity sensor. **c** Fabrication process for the nanoforest-based humidity sensor

The left image in Fig. 1a shows the structures of a traditional PI-based humidity sensor (and the inset shows the structural details of the IDEs). As this image illustrates, a traditional sensor is composed of a substrate, IDEs and a patterned PI layer. The theoretical capacitance model of this sensor is shown in the right image of Fig. 1a, and according to this model, the capacitance of the device is the sum of the capacitances of three capacitors^36,37^,

1 $${C}_{{\rm{T}}}={C}_{{\rm{PI}}}+{C}_{{{\rm{SiO}}}_{2}}+{C}_{{\rm{Si}}}$$

where *C*_PI_ represents the capacitance of the humidity-sensitive layer, which is given theoretically as

2 $${C}_{\text{PI}}=\frac{n{{\rm{\varepsilon }}}_{\text{PI}}{h}_{\text{Finger}}}{{W}_{\text{Gap}}}+\frac{n{l}_{\text{Finger}}{{\rm{\varepsilon }}}_{\text{PI}}}{2}$$

where *n* is the number of IDEs, *l*_Finger_ and *h*_Finger_ are the length and thickness of the IDEs, respectively, and *W*_Gap_ represents the distance between two adjacent electrodes. *ε*_PI_ represents the dielectric constant of the PI layer, which is a variable value that changes with the RH of the environment, as shown in Eq. (3) and Eq. (4):

3 $${{\rm{\varepsilon }}}_{\text{PI}}={[{\rm{\gamma }}({\varepsilon }_{1}^{1/3}-{\varepsilon }_{2}^{1/3})+{\varepsilon }_{2}^{1/3}]}^{3}$$

where *ε*_1_ and *ε*_2_ are the dielectric constants of water and PI, respectively, and *γ* is the fractional volume of water molecules in the PI.

4 $${\rm{\gamma }}={\gamma }_{\text{m}}\exp [-{(RT\mathrm{ln}x/E)}^{b}-\alpha (T-298)]$$

where *γ*_m_ is the maximum fractional volume of absorption at 298 K, *R* is the universal gas constant, *T* is the absolute temperature, *x* is the RH, *α* is the thermal coefficient of limiting absorption, *E* is the free energy of absorption, and *b* is an empirical factor that is determined via trial and error^38^.

Additionally, ${C}_{{{\rm{SiO}}}_{2}}$ is the parasitic capacitance generated by the electric field lines passing through the SiO_2_ layer:

5 $${C}_{{{\rm{SiO}}}_{2}}=\frac{n{l}_{{\rm{Finger}}}{\varepsilon }_{{{\rm{SiO}}}_{2}}}{2}{\beta }_{1}$$

where *ε*_SiO2_ is the dielectric constant of the SiO_2_ layer and *β*_1_ is the proportional factor for the electric field lines after optimized allocation of these lines between the PI layer, the silicon dioxide layer and the silicon substrate.

Furthermore, *C*_Si_ is the parasitic capacitance caused by the field lines passing through the silicon substrate:

6 $${C}_{{\rm{Si}}}=\frac{n{l}_{{\rm{Finger}}}{\varepsilon }_{{\rm{Si}}}}{2}{\beta }_{2}$$

where *ε*_Si_ is the dielectric constant of the silicon substrate and *β*_2_ is the proportional factor of the electric field lines after optimized allocation of the lines between the PI layer, the silicon dioxide layer and the silicon substrate.

Therefore, the existence of these parasitic capacitances (${C}_{{{\rm{SiO}}}_{2}}$ and *C*_Si_) results in the inaccurate determination of the humidity-sensitive layer capacitance (*C*_PI_) and thus also influences the sensitivity of the sensor.

Furthermore, for this type of conventional sensor, two more disadvantages must be considered. The first is the sensor’s inability to minimize the effects of water vapor condensation, which causes longer recovery times. As a result, this disadvantage means that these sensors cannot be used in environments with rapidly changing humidity. The second disadvantage is that temperature drift is not considered in the sensor system, which leads to imprecise humidity sensing.

To address these issues, a novel humidity sensor is designed in this work, as illustrated in Fig. 1b. This device is composed of three layers that contain a humidity-sensing capacitor, a reference capacitor, a microheater and a thermistor. The humidity-sensing capacitor and the reference capacitor are located in the top of the device with consistent IDE structures, thus forming the top layer. The microheater and the thermistor are located in the bottom layer. In between these layers, a silicon dioxide film is inserted to act as an insulation layer, thus forming the intermediate layer.

The theoretical capacitance model of this device is illustrated in the right image of Fig. 1b, and the differential capacitance of the novel configuration is defined as:

7 $${C}_{{\rm{N}}}={C}_{{\rm{H}}}-{C}_{{\rm{R}}}$$

where *C*_H_ is the capacitance of the humidity-sensing capacitor and *C*_R_ is that of the reference capacitor. As illustrated in Fig. 1b, *C*_1_ and *C*_2_ can be expressed as:

8 $${C}_{{\rm{H}}}={C}_{{\rm{NF}}}+{C}_{{\rm{P1}}}+{C}_{{\rm{P2}}}$$

9 $${C}_{{\rm{R}}}={C}_{{{\rm{Si}}}_{3}{{\rm{N}}}_{4}}+{C}_{{\rm{P1}}\text{'}}+{C}_{{\rm{P2}}\text{'}}$$

Here, *C*_NF_ is the capacitance of the nanoforest layer, and *C*_P1_ and *C*_P2_ are the parasitic capacitances generated by the electric field lines passing through the substrate and the silicon dioxide layer, respectively. Similarly, ${C}_{{{\rm{{{Si}_{3}}{N}_4}}}}$ *C*_Si3N4_ is the capacitance of the Si_3_N_4_ layer, and *C*_P1’_ and *C*_P2’_ are the parasitic capacitances in the reference capacitor (corresponding to *C*_P1_ and *C*_P2_ in the sensing capacitor, respectively). With this design, the differential capacitance (*C*_*N*_) is not related to the parasitic capacitance (because *C*_P1_ + *C*_P2_ − *C*_P1’_ − *C*_P2’_ ≈ 0). In addition, the basic differential capacitance of the sensor can be adjusted by varying the structural parameters of the reference capacitor. In this design, a microheater is used to reduce the device recovery time, while a thermistor (*R*_t_) is integrated into the device beside the heater to calibrate the temperature drift and monitor the microheater’s working state. The microheater and the thermistor are both designed to be serpentine to provide uniform heating and high resistance. The ground electrode is set to prevent electromagnetic interference in practical applications, while the electrode surfaces in the device are designed to have large-scale roughness to assist with wire bonding.

The fabrication process of this humidity sensor is illustrated in Fig. 1c. First, a SiO_2_ layer was deposited on a Si substrate; then, a 0.4-μm-thick Al layer was sputtered and patterned to form the microheater, the thermistor and their ground electrodes. A 2-μm-thick SiO_2_ film was subsequently deposited on the Al layer to act as an insulating layer, on which contact holes were then patterned. Subsequently, another Al layer with a thickness of 2 μm was sputtered and patterned to form the IDEs and the top electrodes. The top and ground electrodes were connected via the contact holes. Later, a 2-μm-thick Si_3_N_4_ film was deposited and patterned on one group of IDEs, thus forming the reference capacitor. Subsequently, a PI layer with a thickness of ~8 μm was spin coated onto the other group of IDEs and patterned. Finally, a reactive ion etching (RIE) process using oxygen (O_2_) plasma was used to bombard the PI pattern and form the nanoforest-based sensing capacitor. During the etching step, the applied radio-frequency (RF) power was 200 W, and the O_2_ flow rate was 50 sccm. The O_2_ plasma treatment period was 30 min. Using the process described above, the nanoforest-based triple-layer humidity sensor was realized.

### Structural and morphological characteristics

**Fig. 2:**
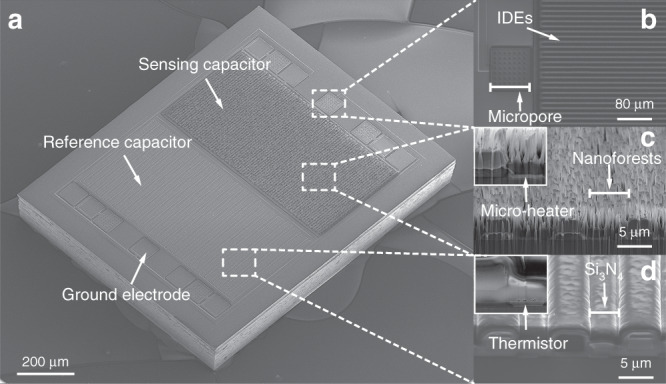
SEM images of the triple-layer humidity sensor. **a** The novel humidity sensor; **b** The IDEs; **c** The sensing capacitor, where the inset shows the microheater below the nanoforests; and **d** The reference capacitor, where the inset shows the thermistor located below the dielectric material

Figure 2 shows scanning electron microscopy (SEM) images of the triple-layer humidity sensor. The dimensions of the humidity sensor are 1100 μm × 890 μm, and the areas of the nanoforest and the Si_3_N_4_ layer are exactly the same (430 μm × 820 μm). SEM images of the nanoforest and the results of the hydrophilicity testing (the contact angle for a water droplet on the nanoforests is approximately 1°) are presented in the Supplementary Information (Fig. S1c). A ground electrode with an array of small holes that is used to assist wire bonding is shown in Fig. 2b. Figure 2c, d shows SEM images of the three layers. A nanoforest (approximately 4 μm high) prepared on the sensing capacitor and a microheater with a width of 4 μm, which is located below the nanoforests, are shown in Fig. 2c. Figure 2d shows the Si_3_N_4_ dielectric layer of the reference capacitor and the thermistor, which has the same structure as the microheater.

For the experiments, four different samples, designated Sample-1 through Sample-4, were prepared. Sample-1, Sample-2 and Sample-3 are three sensors based on nanoforests that have 40, 30 and 20 pairs of IDEs, respectively. Sample-4 is a PI-based sensor that has 40 pairs of IDEs.

### Humidity-sensing properties

**Fig. 3:**
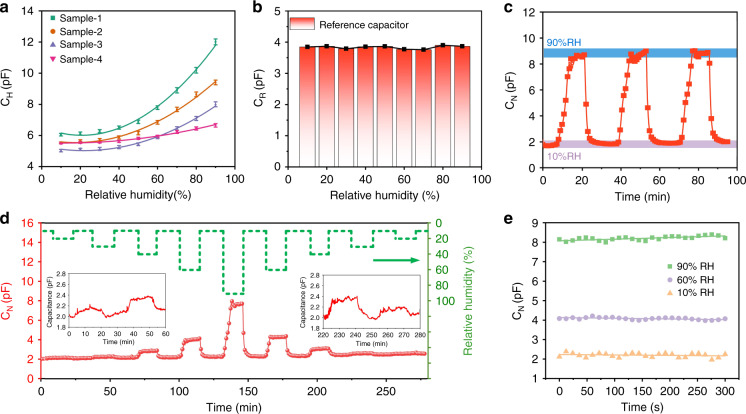
Humidity-sensing properties of the nanoforest-based humidity sensors. **a** Humidity sensing performances of the four humidity sensors. **b** Performance of the reference capacitor in Sample-1. **c** Dynamic humidity-sensing response of Sample-1 under different humidity conditions. **d** Continuous humidity sensing curve for Sample-1. **e** Stability of the humidity sensitivity of Sample-1 under different RH conditions

The humidity-sensing properties of the nanoforest-based humidity sensors are illustrated in Fig. 3. As Fig. 3a shows, the capacitance of each of the four different sensors increases with increasing RH, and their sensitivity also increases with increasing numbers of IDEs (at 20 °C). The sensing capacitor (*C*_H_) in Sample-1 has the highest sensitivity of 0.11 pF/%RH at 40–90% RH (Fig. S4), which is approximately 8 times higher than that of Sample-4 (0.014 pF/%RH). In addition, the resolutions of the sensor are 0.72% RH and 0.075% RH at 10–40% RH and 40–90% RH, respectively. Because Sample-1 is the most sensitive of the four sensors, this device was used for further investigations. As illustrated in Fig. 3b, the reference capacitance (*C*_R_) of this device remains stable (at approximately 3.85 pF) under different RH conditions, thus meaning that the differential capacitance (*C*_N_ = *C*_H_ − *C*_R_) can be used in this sensor. The following capacitance response results were all processed using the difference method.

In practical applications of humidity sensors, repeatability is a critical performance indicator. As shown in Fig. 3c, the continuous response and recovery curves of Sample-1 at 10% RH and 90% RH were stable, and the humidity-sensing response at 10% RH was maintained at 1.92 pF, while the corresponding response at 90% RH was 8.15 pF. These results demonstrate that the nanoforest-based humidity sensor provides excellent repeatability and stability under different RH conditions. Figure 3d shows the real-time capacitance variations of the device when the RH conditions varied from 10% to 90% (the inset shows the capacitance response curves of the humidity sensor from 10% RH to 40% RH and 40% RH to 10% RH). These results demonstrate that the nanoforest-based humidity sensor provides perfect sensitivity over a wide RH range. Figure 3e shows the stability of the humidity sensitivity of Sample-1.

**Fig. 4:**
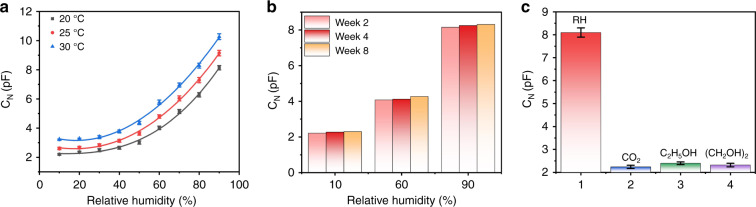
Temperature drift, long-term stability and selectivity test of the nanoforest-based humidity sensor. **a** Humidity response curves for the sensor at different temperatures. **b** Long-term stability of the sensor. **c** Gas selectivity of the sensor

Temperature drift is another important performance characteristic for humidity sensors. Figure 4a shows the capacitance curves for the sensor at three different temperatures. As the curves show, the device maintains high sensitivity at different temperatures with a positive temperature coefficient. It is known that relative humidity is the ratio of the absolute humidity in the air to the saturated absolute humidity at the same temperature; a higher temperature leads to higher saturated absolute humidity, which represents the maximum number of water molecules that can be contained per unit volume of air at this temperature. Therefore, under the same relative humidity conditions, there are more water molecules at a higher temperature, which results in more water molecules being absorbed by the humidity-sensitive material. Therefore, for most capacitance-based humidity sensors, there will be an increment in capacitance when the temperature is rising. Figure 4b illustrates the long-term stability of the sensor. The results demonstrate that the capacitance response of the sensor remained stable over 8 weeks of monitoring.

Humidity sensors are often used in complex gas environments, and the sensor specificity is thus particularly important. To verify the specificity of this type of sensor, its response was tested in different gas environments containing carbon dioxide (100 ppm), ethylene glycol (500 ppm) and ethanol (100 ppm), and the results were compared with the sensor’s response at 90% RH. As Fig. 4c illustrates, the humidity sensor showed little response to these other gases, thus verifying the good selectivity of the sensor.

### Temperature-sensing properties

**Fig. 5:**
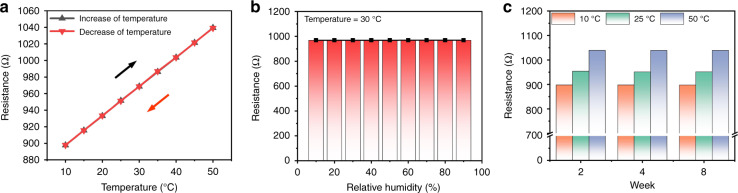
Temperature-sensing properties of the thermistor. **a** Hysteresis curve of the thermistor. **b** Resistance of the thermistor under various humidity conditions. **c** Long-term stability of the thermistor

Because aluminum has a positive temperature coefficient, the resistance of the aluminum-based thermistor increases with increasing temperature. In the experiment, the chamber was set to different temperatures (initially increasing from 10 °C to 50 °C with a step of 5 °C and returning to 10 °C with the same step size), and the resistance of the thermistor varied accordingly between 897.7 Ω and 1039.3 Ω, showing a linear tendency (Fig. 5a). These results indicate that the thermistor performance shows excellent linearity within the range from 10 °C to 50 °C.

Furthermore, when the RH increases from 10% to 90% at 30 °C, the resistance of the thermistor also remains stable (Fig. 5b). This indicates that the thermistor’s resistance is not affected by the RH. Figure 5c shows the long-term stability characteristics of the thermistor. In practical applications, the ambient temperature could be detected using the thermistor before taking humidity measurements, thus enabling the temperature drift issue to be addressed; this could greatly enhance the accuracy of the measured humidity value and expand the potential application fields of the sensor.

### Microheater performance

**Fig. 6:**
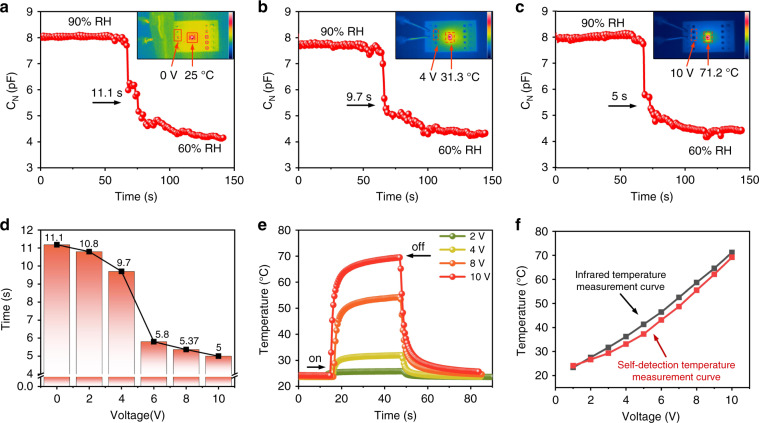
Performance test of microheater. **a**–**c** Recovery curves for the sensor at different heating temperatures; the insets show infrared images of the microheater at different voltages. (**d**) Recovery times of the humidity sensor when the microheater is operating at different voltages. **e** Response time curves of the thermistor when the microheater is operational. **f** Infrared temperature measurement curve and self-detection temperature measurement curve of the microheater

The evaporation rate of water is largely dependent on the surrounding temperature. When the microheater is operating within the sensor, the surrounding temperature of the sensor will then increase to a stable value, which is further determined by the voltage applied to the sensor. The relationship between the applied voltage and the recovery time for the sensor is illustrated in Fig. 6a–d. When the micro-heater was not operating (i.e., when the applied voltage was 0 V and the surrounding temperature was 25 °C), the recovery time was approximately 11.1 s (Fig. 6a). However, when the applied voltage was increased to 4 V (and the temperature surrounding the sensor was increased to 31.3 °C), the recovery time was reduced to 7.7 s (Fig. 6b), and the time was reduced further to only 5 s when the applied voltage reached 10 V. In this state, the temperature of the microheater rose to 71.2 °C (Fig. 6c). The relationship between the operating voltage and the recovery time is shown in detail in Fig. 6d. The sensor recovery time is reduced by 6.1 s through the use of the microheater. However, the shortening of the recovery time is also limited by the response time of the microheater.

The response time and the working temperature of the microheater can be monitored using the thermistor. As shown in Fig. 6e, the microheater’s response time remained at approximately 4.7 s when several different voltages (2 V, 4 V, 8 V and 10 V) were applied to the microheater. However, as shown in Fig. 6f, the thermistor-detected temperatures showed few differences compared to those obtained from the infrared imaging results. There is a small deviation (the maximum deviation is 3.6 °C) caused by the heat lost from the device surface via heat conduction. However, the microheater operates at 10 V, which means that this deviation does not affect the rough estimate of the working temperature.

### Human respiratory monitoring

**Fig. 7:**
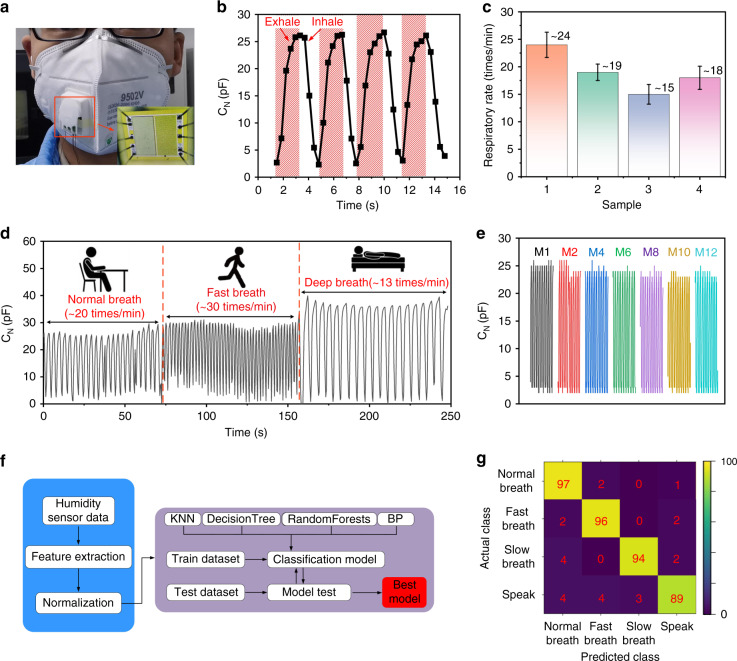
Human respiratory monitoring. **a** Photograph showing the humidity sensor in a mask worn by a volunteer. **b** Capacitance response curve during breathing (four cycles). **c** Respiratory monitoring results for four randomly selected volunteers. **d** Responses of the sensor to the different respiratory modes. **e** Respiratory response curves recorded for 12 months from the same device. **f** Flow chart for the respiratory state recognition algorithm based on machine learning. **g** Classification test confusion matrix with 400 groups of the dataset during recognition of four different respiratory states. The color bar represents the sizes of the predicted numbers

The respiratory monitoring process mainly includes respiratory depth monitoring and respiratory frequency monitoring. Different respiratory states are closely related to different respiratory diseases^39–41^. From this perspective, human respiratory monitoring is of interest for both consumer applications (e.g., exercise monitoring) and medical diagnosis (e.g., sleep apnea detection)^42,43^. In the experiments, we placed our humidity sensors inside several masks with breathing holes. Then, data were recorded at different respiratory states when volunteers were wearing these masks (as shown in Fig. 7a). During the exhalation process, when the gas flow is highly moisturized (compared with the RH of the environment), the water vapor adheres to the surface of the nanoforests, thus resulting in a significant increase in capacitance. This sensor can thus be used for human respiratory monitoring while ignoring the effects of environmental humidity fluctuations. In contrast, during the inspiration process, the external gas flow from the surrounding environment removes the water vapor from the device surface, meaning that the relative humidity level around the sensor is approximately the same as that of the surrounding environment. The capacitance variations in the sensor during four complete respiration cycles are illustrated in Fig. 7b, and the results obtained for the simulated apnea syndrome are shown in Fig. S8. These results confirm that the sensor can successfully track every breath of the volunteer.

The nonobstructive feature of the proposed sensor allows continuous in situ monitoring when used as a wearable device. Therefore, this type of humidity sensor can be used to record respiratory depths and frequencies for people with different respiratory states. For an adult, the duration of a typical respiration cycle is 3–4 s, but different people may have different breathing frequencies. In our experiment, four healthy volunteers were selected, and the same humidity sensor was inserted into four masks to monitor the respiratory rates of these volunteers (detailed data are shown in the Supplementary Information (Fig. S8)). Figure 7c shows the monitoring results, which are 24, 19, 15 and 18 times per minute for the four volunteers.

Figure 7d shows the capacitance variations of volunteer no. 2 in the three respiratory modes recorded by the sensor, which are the deep breathing mode (~13 times/min, e.g., during sleep or yoga), the normal mode (~20 times/min, e.g., when reading or studying) and the fast mode (~30 times/min, e.g., during walk or exercise). The results illustrate that the sensor can distinguish the status of a body by detecting variations in respiratory rates and depths. Furthermore, because respiratory monitoring requires the humidity sensor to operate in a high humidity environment for long periods, its long-term stability is extremely important. As illustrated in Fig. 7e, the respiratory monitoring signals from the sensor remained stable after use for 12 months.

Machine learning plays an important role in the sensing field due to its powerful data analysis and mining capabilities. In this work, humidity testing data were analyzed using machine learning algorithms to improve the accuracy of respiratory state prediction for volunteers. The flow chart for the respiratory state recognition algorithm based on machine learning is shown in Fig. 7f. The humidity testing data were recorded from experiments involving volunteers. These data were then divided into a training dataset (1600 sets) and a testing dataset (400 sets). In the training dataset, there were 400 sets for each of the four states, including normal breathing, fast breathing, deep breathing and speaking. In addition, in the training dataset, there were 100 sets for each of the four states. These datasets were imported into the Python 3.8 development environment for feature extraction, normalization and model testing using the Numpy library and Scikit-learn library. To determine the best prediction model for each respiratory state, four different algorithms, including the K-nearest neighbor (KNN), decision tree, random forest and backpropagation neural network (BPNN) algorithms, were used. The recognition accuracy rates for the respiratory state recognition algorithms are shown in the Supplementary Information (Fig. S11). Among these algorithms, the BPNN algorithm showed the highest accuracy rate of up to 94% (the detailed classification accuracy for the four different states based on the BPNN algorithm are shown in Fig. 7g), which indicates that the use of the sensor in consumer electronics and intelligent medical instrument applications is feasible.

### Sensing mechanism

**Fig. 8:**
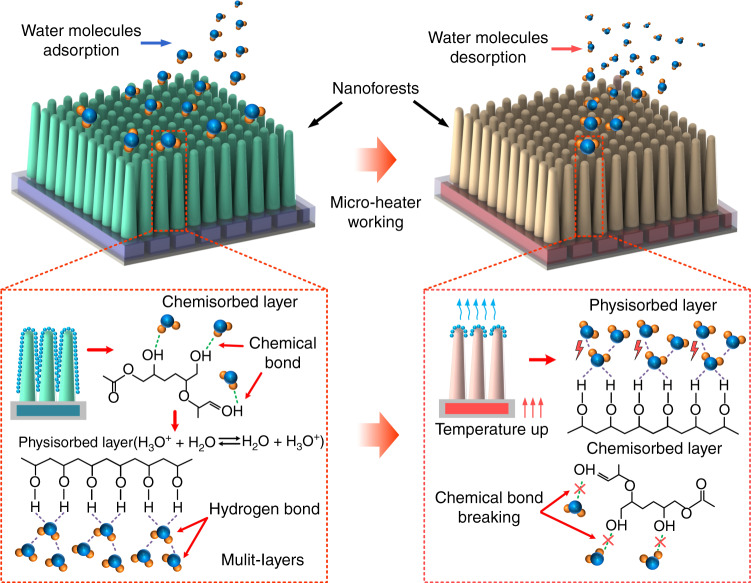
Humidity-sensing mechanism of the nanoforests. Schematic diagram of the water molecules adsorption (left) and desorption (right) on the surface of the nanoforests

Figure 8 is a schematic diagram showing the mechanism of the nanoforests used for the humidity sensing. In the low humidity range of 10–40%RH, a small number of water molecules are more likely to be chemisorbed at the active sites (hydrophilic carboxyl (-COOH) and hydroxyl (-OH)) around the nanoforests through hydrogen bonds, which causes only a capacitance increase of 1 pF. When the RH increases to a certain extent, the chemisorption active sites are all occupied. Then, with the increase in RH, physisorption takes place and begins to create a layer of water molecules, which are weakly bonded by van der Waals interactions. As the humidity level rises further, more water molecules are adsorbed by this first physisorption layer through hydrogen bonding, thereby forming the second through the *n*^th^ layers of the water molecules, which, as a result, causes a 5 pF increase in capacitance. Based on the synergy between chemisorption and physisorption, the relationship between relative humidity and capacitance obtained experimentally in Fig. 3a is nonlinear, and there is an obvious critical point at 40% RH. In summary, the measurement results validated this sensing mechanism.

In contrast, during the desorption process, when the microheater is operating, the temperatures of the nanoforest and the surrounding environment are increased. The movement of the water molecules thus becomes more vigorous, and both the chemical and hydrogen bonds breakdown more rapidly, which results in the faster evaporation of water molecules. As a result, the sensor’s recovery time is only 5 s, which is much shorter than that of previously reported capacitive and resistive humidity sensors, as listed in Table. S1 in the supplementary information.

## Conclusion

In summary, we have proposed a novel humidity sensor that contains a sensing capacitor, a reference capacitor, a thermistor and a microheater to improve the sensing performance. When compared with traditional PI-based humidity sensors, the sensitivity of the proposed device with in situ integration of the nanoforests is enhanced significantly. In addition, by adding a microheater, a thermistor and a reference capacitor to the sensor, the proposed humidity device gains the ability to self-improve its signal accuracy and self-reduce its response time, in addition to its self-detection ability. Theoretical analyses and experimental tests were performed to investigate the effects of the number of IDEs and the heating temperature on the humidity sensing performance, and the advantages of the difference method used in the device were demonstrated. Based on the excellent performance demonstrated by the sensor and by using machine learning algorithms, we used the humidity sensor for the respiratory monitoring of human breathing. It is expected that such a highly sensitive humidity sensor will be used widely in the future in a variety of commercial applications.

## Materials and methods

### Materials

PI (ZKPI-5100) was purchased from POME Technology, Beijing, China. The gases (CO_2_, N_2_) used in the device calibration process were stored in steel cylinders purchased from Guangyun Gas Co., Ltd., Wuxi, China. The ethylene glycol and ethanol used in the experiments were of analytical purity and were purchased from Sinopharm Chemical Reagent Co., Ltd., Shanghai, China. The masks used for the insertion of the sensors were purchased from 3 M China.

### Characterization and test methods

A high-resolution scanning electron microscope (GeminiSEM 300, Carl Zeiss, Jena, Germany) and a focused ion beam (FIB) system (Helios G4 CX, Thermo Fisher Scientific, Waltham, USA) were used to investigate the humidity sensor structures. The humidity sensor was secured in a window-type TO package that was then connected to a printed circuit board layer through wire bonding. To evaluate the humidity-sensing capability of the sensor, it was placed in the chamber of a temperature-humidity generator (HG2-S, Rotronic, Switzerland). Capacitance signals were collected using a high-precision multimeter (DMM6500, Keithley, USA). The working temperatures of the microheater at different voltages were tested using an infrared imaging measurement system (MicroOptics Research IR, Beijing, China). The working voltage for the microheater was applied using a DC power supply (Victor 3005 A, China). To evaluate the specificity of the humidity sensor in different gas environments, the sensor was placed in a chamber in which the gas concentrations could be controlled (DGL-3, ELITE TECH, China).

## Supplementary information

Supplementary information of MICRONANO-01916R1

## References

[1] Yang J, Shi R, Lou Z, Chai R, Shen G (2019). Flexible smart noncontact control systems with ultrasensitive humidity sensors. Small.

[2] Kano S, Kim K, Fujii M (2017). Fast-response and flexible nanocrystal-based humidity sensor for monitoring human respiration and water evaporation on skin. ACS Sens.

[3] Dai J (2019). Ultrafast response polyelectrolyte humidity sensor for respiration monitoring. ACS Appl. Mater. Inter..

[4] Lu L, Jiang C, Hu G, Liu J, Yang B (2021). Flexible noncontact sensing for human–machine interaction. Adv. Mater..

[5] Kim KH, Kim HD (2020). Deep sleep mode based node MCU-enabled humidity sensor nodes monitoring for low-power IoT. J. Mater. Sci. Mater. Electron.

[6] Zhou L (2021). High-performance humidity sensor based on graphitic carbon nitride/polyethylene oxide and construction of sensor array for non-contact humidity detection. Sens. Actuat. B Chem..

[7] Delannoy FP, Sorli B, Boyer A (2000). Quartz crystal microbalance used as humidity sensor. Sens. Actuat. A Phys..

[8] Iyengar SA, Srikrishnarka P, Jana SK, Islam MR, Pradeep T (2019). Surface treated nanofibers as high current yielding breath humidity sensors for wearable electronics. ACS Appl. Electron. Ma.

[9] Le X, Liu Y, Peng L, Pang J, Xie J (2019). Surface acoustic wave humidity sensors based on uniform and thickness controllable graphene oxide thin films formed by surface tension. Microsyst. Nanoeng..

[10] Wooseong (2019). Breathable nanomesh humidity sensor for real-time skin humidity monitoring. ACS Appl. Mater. Inter..

[11] Santha H, Packirisamy M, Stiharu I, Li X, Rinaldi G (2005). A polyimide based resistive humidity sensor. Sens. Rev..

[12] Dai CL (2007). A capacitive humidity sensor integrated with micro heater and ring oscillator circuit fabricated by CMOS-MEMS technique. Sens. Actuat. B Chem..

[13] Yeow J, She J (2006). Carbon nanotube-enhanced capillary condensation for a capacitive humidity sensor. Nanotechnology.

[14] Wang Q, Lian M, Zhu X, Chen X (2021). Excellent humidity sensor based on ultrathin HKUST-1 nanosheets. RSC Adv..

[15] Li Z, Dong B, Chen E, Li Y, Gao C (2021). High sensitivity FBG humidity sensor coated with graphene and polyimide films. Opt. Fiber Technol..

[16] Fan X, Wang Q, Zhou M, Liu F, Meng H (2020). Humidity sensor based on a graphene oxide-coated few-mode fiber Mach-Zehnder interferometer. Opt. Express.

[17] Lazarus N, Sarah S. Bedair, Lo CC, Fedder GK (2010). CMOS-MEMS capacitive humidity sensor. J. Microelectromech Syst..

[18] Wu J, Wu Z, Ding H, Wei Y, Wang X (2019). Multifunctional and high-sensitive sensor capable of detecting humidity, temperature, and flow stimuli using an integrated microheater. ACS Appl. Mater. Inter..

[19] Liu RW, Tai YL (1996). A polymer resistive-type humidity sensor. J. Wenzhou Teach. Coll..

[20] Fuerjes P (2003). Porous silicon-based humidity sensor with interdigital electrodes and internal heaters. Sens. Actuat. B Chem..

[21] Zhang Y (2005). Zinc oxide nanorod and nanowire for humidity sensor. Appl. Surf. Sci..

[22] Chen HJ, Xue QZ, Ma M, Zhou XY (2010). Capacitive humidity sensor based on amorphous carbon film/n-Si heterojunctions. Sens. Actuat. B Chem..

[23] Kim Y (2009). Capacitive humidity sensor design based on anodic aluminum oxide. Sens. Actuat. B Chem..

[24] Wang Q (2011). Resistive and capacitive response of nitrogen-doped TiO_2_ nanotubes film humidity sensor. Nanotechnology.

[25] Wei Y, Chen X, Jian Z (2010). A capacitive humidity sensor based on gold–PVA core–shell nanocomposites. Sens. Actuat. B Chem..

[26] Schubert PJ, Nevin JH (1985). A polyimide-based capacitive humidity sensor. IEEE Trans. Electron. Devices.

[27] Chen, S. C., Chung, V., Yao, D. J. & Fang, W. Vertically integrated CMOS-MEMS capacitive humidity sensor and a resistive temperature detector for environment application. *Transducers*, 1453–1454 (2017).

[28] Rittersma ZM, Splinter A, B. Decker A, Benecke W (2000). A novel surface-micromachined capacitive porous silicon humidity sensor. Sens. Actuat. B Chem..

[29] Zhu Z, Yang G, Li R, Pan T (2017). Photopatternable PEDOT:PSS/PEG hybrid thin film with moisture stability and sensitivity. Microsyst. Nanoeng..

[30] Gerwen PV (1998). Nanoscaled interdigitated electrode arrays for biochemical sensors. Sens. Actuat. B Chem..

[31] Huang, J. Q., Chen, W. H., Zhu, D. P. & Han, L. A CMOS interdigital capacitive humidity sensor enhanced by a multi-layered structure. *IEEE Sens*., 1459–1462 (2014).

[32] Dai CL, Liu MC, Chen FS, Wu CC, Chang MW (2007). A nanowire WO_3_ humidity sensor integrated with micro-heater and inverting amplifier circuit on chip manufactured using CMOS-MEMS technique. Sens. Actuat. B Chem..

[33] Palma AJ (2014). Design and characterization of a low thermal drift capacitive humidity sensor by inkjet-printing. Sens. Actuat. B Chem..

[34] Chen G (2021). Performance enhanced humidity sensor by in-situ integration of nanoforests. IEEE Electron. Device Lett..

[35] Gu L, Huang QA, Qin M (2004). A novel capacitive-type humidity sensor using CMOS fabrication technology. Sens. Actuat. B Chem..

[36] Sacher E, Susko JR (1979). Water permeation of polymer films. I. Polyimide. J. Appl. Polym. Sci..

[37] Sacher E (1979). J., R. & Susko. Water permeation of polymer films. III. High.-Temp. Polyim. J. Appl. Polym. Sci..

[38] Zhou WH, Wang LC, Wang LB (2016). Numerical study of the structural parameter effects on the dynamic characteristics of a polyimide film micro-capacitive humidity sensor. IEEE Sen. J..

[39] Tai H, Wang S, Duan Z, Jiang Y (2020). Evolution of breath analysis based on humidity and gas sensors: potential and challenges. Sens. Actuat. B Chem..

[40] Yi Y, Jiang Y, Zhao H, Brambilla G, Wang P (2020). High-performance ultrafast humidity sensor based on microknot resonator-assisted machZehnder for monitoring human breath. ACS Sens.

[41] Li X, Zhuang Z, Qi D, Zhao C (2020). High sensitive and fast response humidity sensor based on polymer composite nanofibers for breath monitoring and non-contact sensing. Sens. Actuat. B Chem..

[42] Güntner AT (2019). Breath sensors for health monitoring. ACS Sens.

[43] B HYA (2021). Microwave humidity sensor based on carbon dots-decorated MOF-derived porous Co_3_O_4_ for breath monitoring and finger moisture detection. Carbon.

