# Supplementary material for: A nanoforest-based humidity sensor for respiration monitoring
Source: Microsyst Nanoeng. 2022 Apr 21;8:44. doi: 10.1038/s41378-022-00372-4 (PMC9023489; doi:10.1038/s41378-022-00372-4)
Supplement: Supplementary file 1 — Supplementary information of MICRONANO-01916R1 [file 41378_2022_372_MOESM1_ESM.doc]

**Supplementary Information**

**A nanoforest-based humidity sensor for respiration monitoring**

Guidong Chen^1,2^, Ruofei Guan^3^, Meng Shi^1,2^, Xin Dai^3^, Hongbo Li^1,2^, Na Zhou^1,2*^, Dapeng Chen^1,2,3^ and Haiyang Mao^1,2,3*^

^1^ Institute of Microelectronics of Chinese Academy of Sciences, Beijing 100029, China.

^2^ University of Chinese Academy of Sciences, Beijing 100049, China.

^3^ Jiangsu Hinovaic Technologies Co., Ltd., Wuxi 214135, China.

^*^ Corresponding authors: Haiyang Mao (maohaiyang@ime.ac.cn); Na Zhou (zhouna@ime.ac.cn)

**Contents**

**Figure S1.** Figure S1. SEM images of two humidity sensitive materials.

**Figure S2.** Photograph of the novel humidity sensor.

**Figure S3.** Photograph of the humidity sensor after packaging.

**Figure S4.** The linear fitting curve of the humidity sensor in the RH ranges of 10-40% and 40-90%.

**Figure S5.** Humidity cycling tests for the nanoforest-based humidity sensor at different times.

**Figure S6.** Temperature cycling tests for the nanoforest-based humidity sensor. at different times.

**Figure S7.** Structural stability of the nanoforests after several humidity cycles.

**Figure S8.** Test curve for sleep apnea.

**Figure S9.** Response curves for the breathing of four volunteers.

**Figure S10.** Response curve for volunteer no. 2 for 40 min.

**Figure S11.** Recognition accuracy rates of four different respiratory state recognition algorithms.

**Figure S12.** Classification test confusion matrix with 400 groups of the dataset for recognition of four different respiratory states based on three different algorithms.

**Table S1.** Performance of humidity sensors based on different sensing materials**.**

Figure S1(a) shows an SEM image of a PI layer with pores inside. Figure S1(b) and (c) show SEM images of nanoforests, which is a type of fully-open nanostructure with a height of 4 μm,an average diameter of 100 nm and an inter-distance of 70 nm. These nanostructures provide a super-large specific surface area, which is beneficial for adsorption of water molecules. The inset shown in Fig. S1(c) demonstrates that the water contact angle on these nanoforests is smaller than 1°.


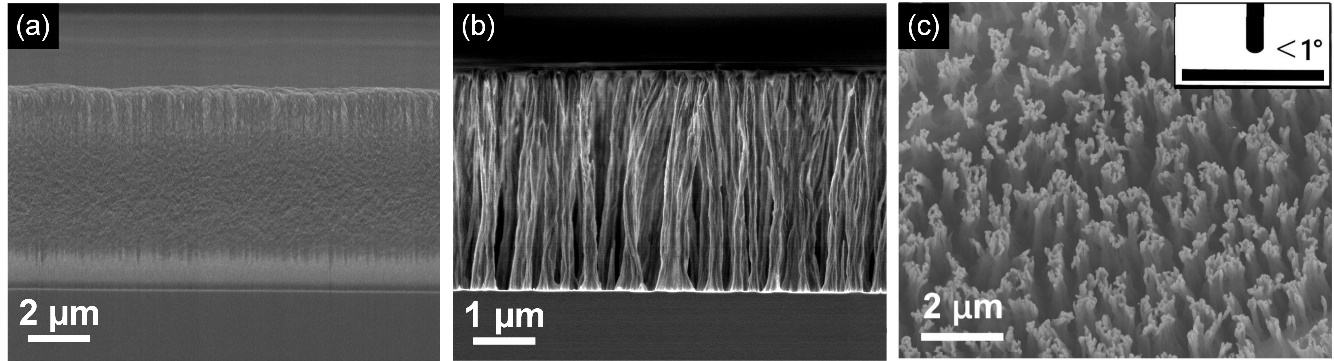
**Figure S1.** **SEM images of two humidity sensitive materials.** (a) PI. (b) and (c) Nanoforests.

Figure S2 shows a photograph of the novel humidity sensor. The sensor dimensions are approximately 1100 µm × 890 µm × 675 µm.


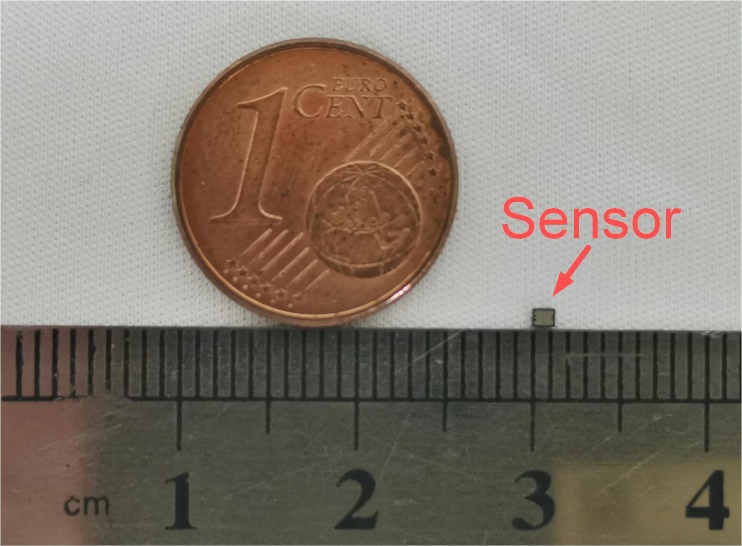


**Figure S2. Photograph of the novel humidity sensor.**

Figure S3 shows a photograph of the sensor after packaging. The sensor is protected by a circular metal casing with two windows and is connected to the bottom printed circuit board (PCB) using gold wires. This package structure has 10 pins that correspond to five functions, where pin 1 is used to test the sensing capacitor, pin 2 is used to test the reference capacitor, pin 3 is used to power the micro-heater, pin 4 is used to test the thermistor, and pin 5 is set as the ground electrode.


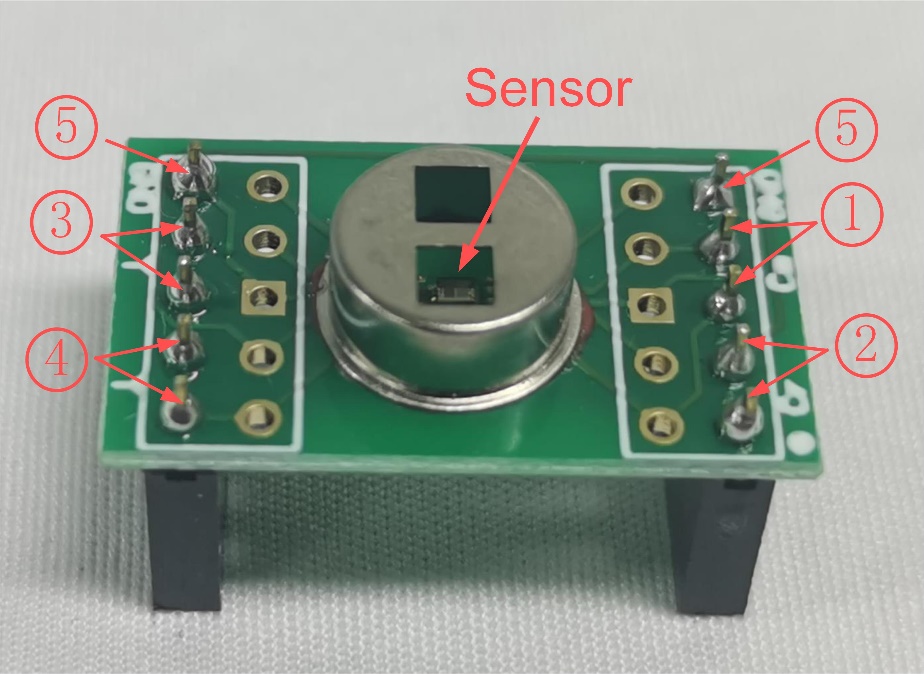


**Figure S3. Photograph of the humidity sensor after packaging.**

We fit the humidity sensitivity curve of the sensor in two separate ranges, namely the 10-40%RH and the 40-90%RH. Figure S4 shows the linear relationship between capacitance and RH obtained by fitting, here we get, *y* = 0.014*x*+5.907 (*R*^2^ = 0.97, 10-40%RH) and *y* = 0.11*x*+1.544 (*R*^2^ = 0.98, 40-90%RH). Accordingly, the sensing capacitor (C_H_) in Sample-1 has a sensitivity of 0.11 pF/%RH in the range of 40-90%RH, and of 0.014 pF/%RH in the 10-40%RH range.


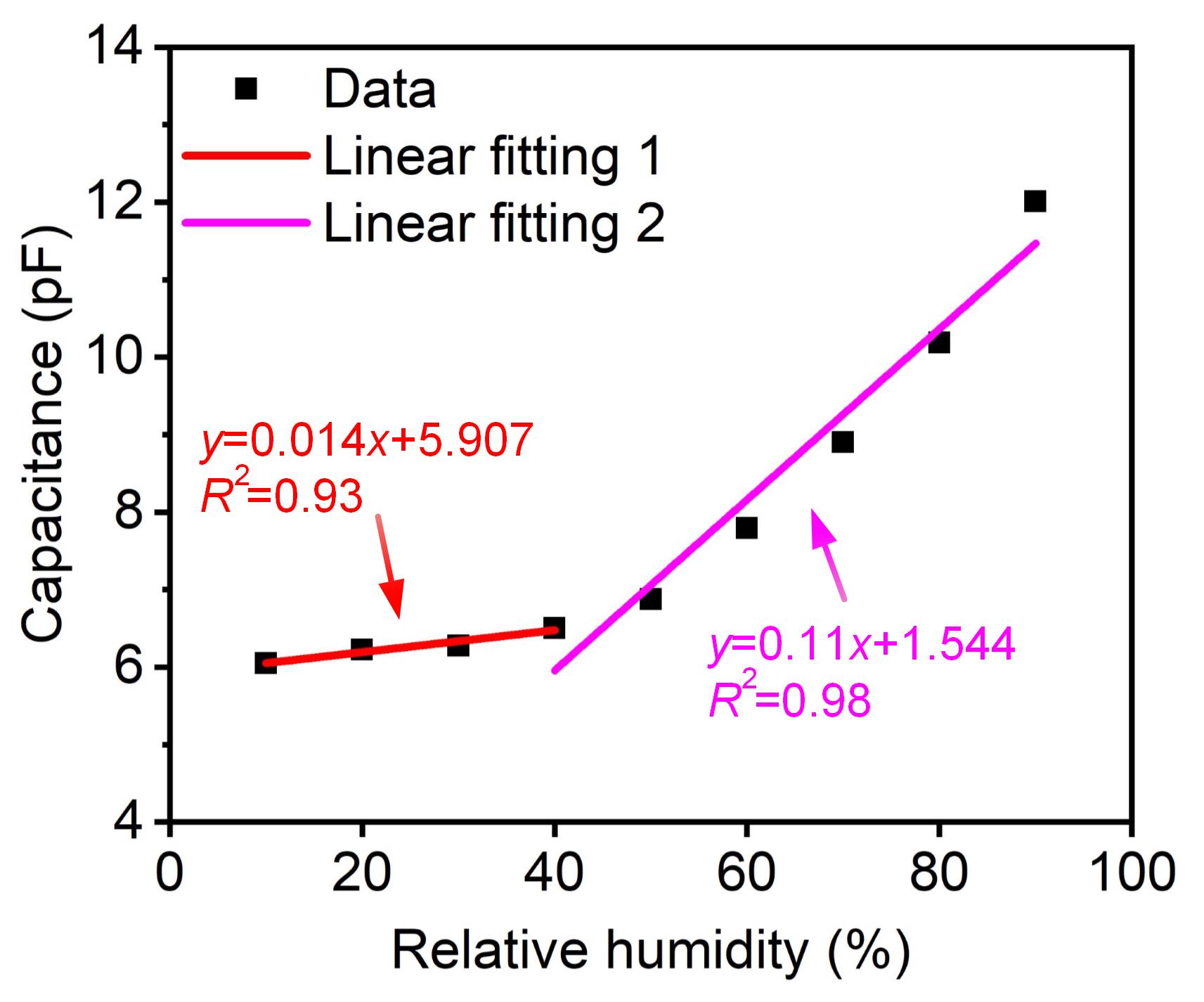


**Figure S4. The linear fitting curve of the humidity sensor in the RH ranges of 10-40% and 40-90%.**

For long-term stability evaluation, humidity cycling experiments were performed for 8 weeks, while the capacitance values of the device were acquired at week 2, 4 and 8. In the experiment, the temperature of the temperature-humidity generator was set at 20 ℃, the device was put in the chamber going through humidity cycles, and at the 2nd, 4th and 8th week, data were collected at 10%RH-60%RH-90%RH-60%RH-10%RH, the results are illustrated in Figure S5. Then in the temperature cycling experiment, the relative humidity of the temperature-humidity generator was set at 50%, the device was tested at 20 ℃-25 ℃- 30 ℃-25 ℃-20 ℃, the results are shown in Figure S6. These results demonstrate that the capacitive response of the sensor remains stable after the device went through long-term cycling of humidity and temperature conditions. These results demonstrate that the capacitive response of the sensor remains stable after the device went through long-term cycling of humidity conditions.

In addition, we also evaluated stability of the nanoforests after several cycles of humidity testing according to the SEM images. As shown Figure S7, morphology variations of the nanoforests are not obviously observed after the humidity cycling. In other words, nanoforests have excellent structural stability.


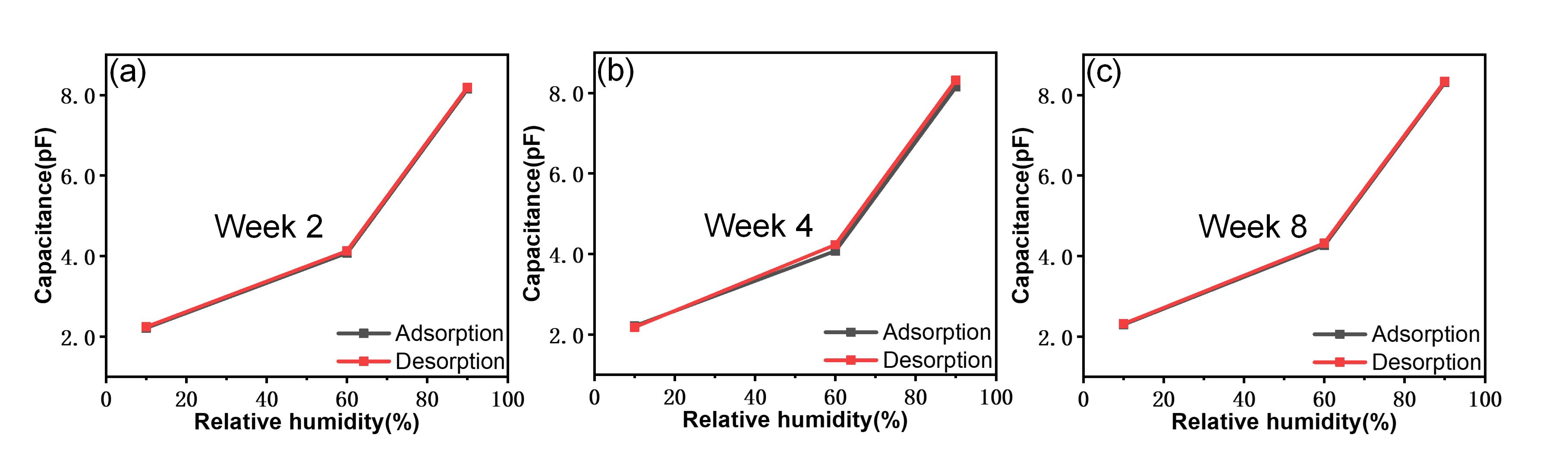


**Figure S5. Humidity cycling tests for the nanoforest-based humidity sensor at different times.** (a) At week 2. (a) At week 4. (a) At week 8.


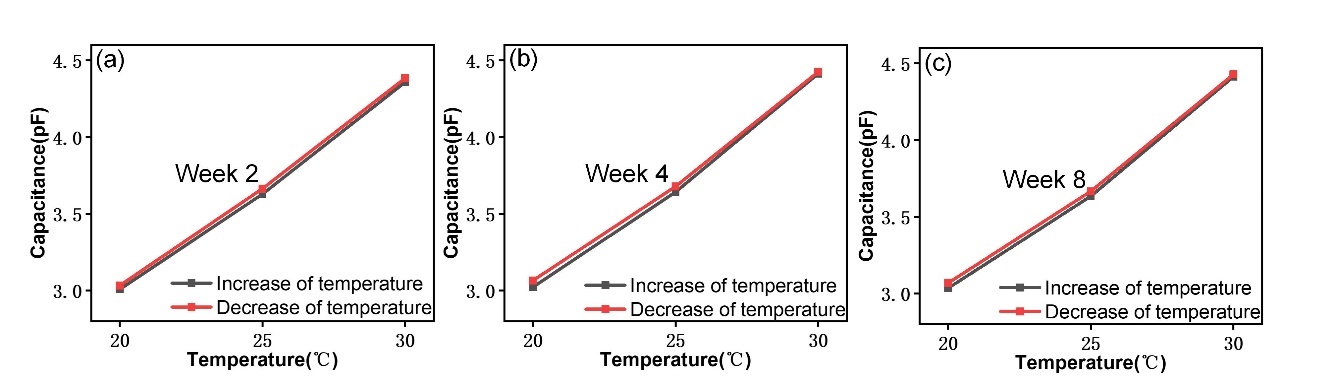


**Figure S6. Temperature cycling tests for the nanoforest-based humidity sensor. at different times.** (a) At week 2. (a) At week 4. (a) At week 8.


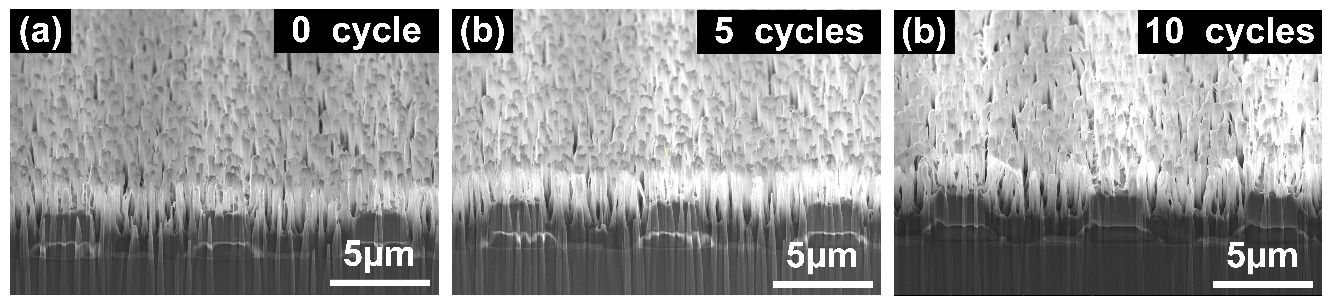


**Figure S7. Structural stability of the nanoforests after several humidity cycles.** (a) 0 cycle. (b) 5 cycles. (c)10 cycles.

Figure S8 shows the test curve for apnea. During breathing monitoring for 90 s, the volunteer simulated sleep apnea syndrome by pausing his breathing three times. The results show that the sensor can clearly track the moment at which pausing begins and the time for which the volunteer’s breath is paused. Therefore, the sensor is expected to be used in medical monitoring system applications.


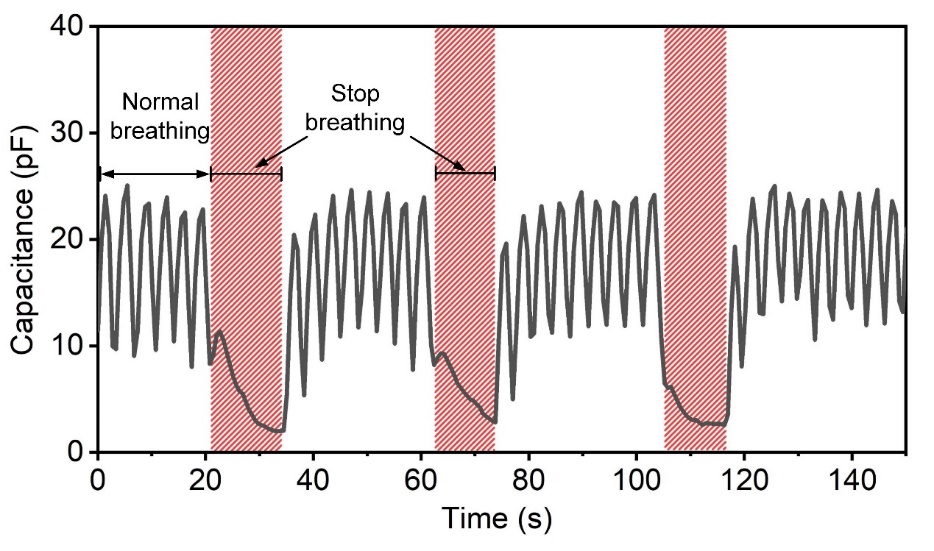


**Figure S8.** **Test curve for sleep apnea.**

Figure S9 shows the response curves for the breathing of four volunteers. Different people have different breathing habits. In future applications, the sensor will need to provide a custom calibration system for the different users.


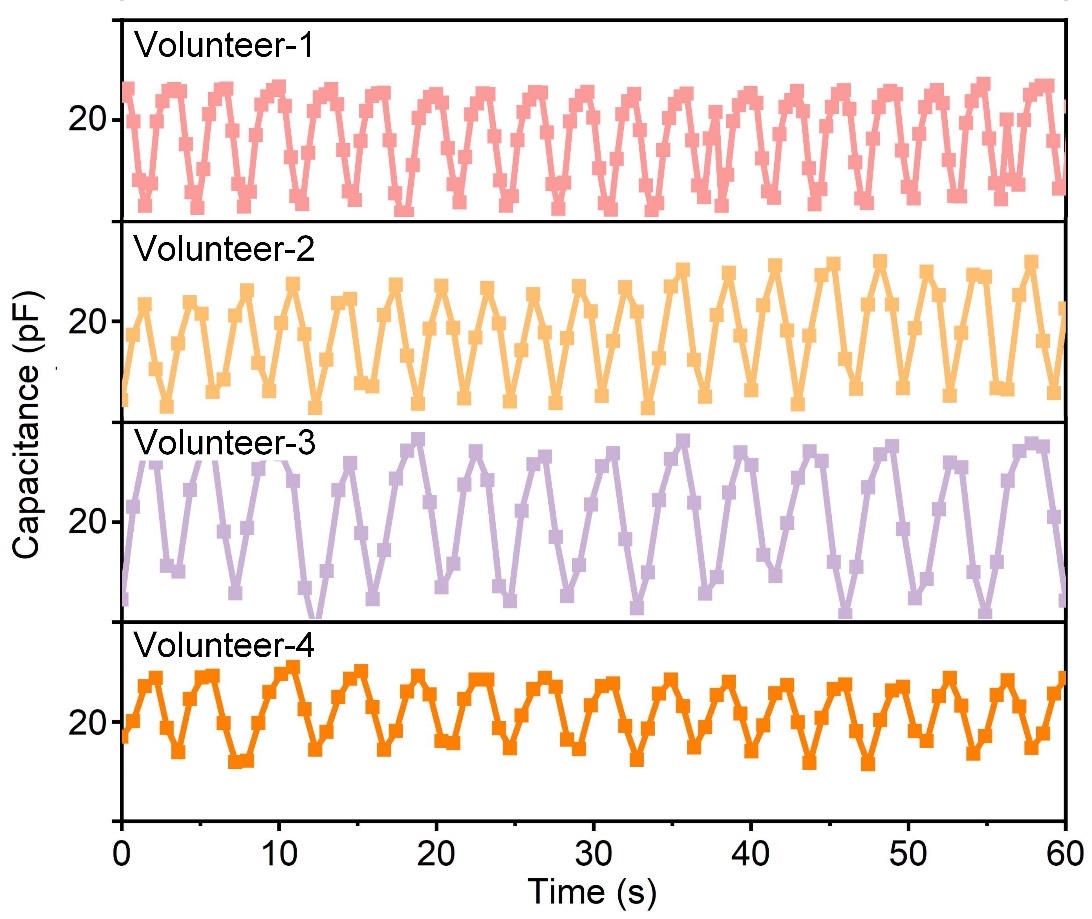


**Figure S9. Response curves for the breathing of four volunteers.**

Figure S10 shows the humidity sensor data that were recorded from volunteer no. 2 for a 40 min period.


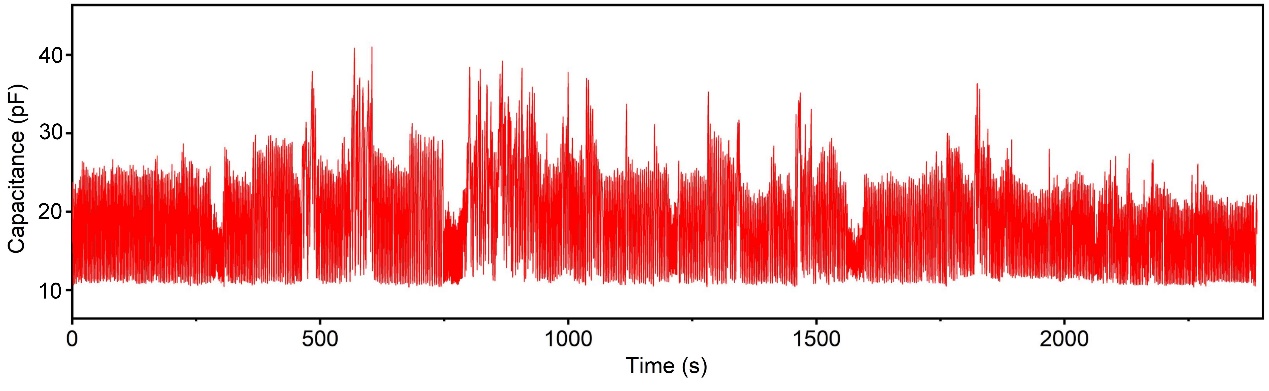


**Figure S10. Response curve for volunteer no. 2 for 40 min.**

Four different algorithms (including K-nearest neighbor (KNN), Decision Tree, Random Forests, and backpropagation (BP) neural network algorithms) were applied. The recognition accuracy rates of the four different respiratory state recognition algorithms are shown in Fig. S11. Among these algorithms, the BPNN algorithm showed the highest accuracy rate up to 94% (the accuracy rates for the KNN, Decision Tree and Random Forests algorithms are 84%, 80% and 85%, respectively). The detailed classification accuracy for the four different states based on KNN, Decision Tree and Random Forests algorithms are shown in Fig. S12.


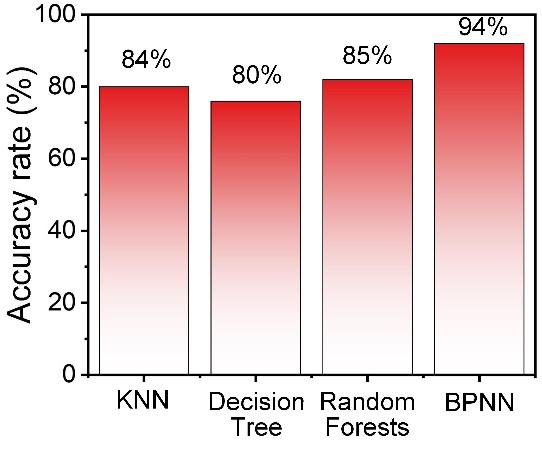


**Figure S11. Recognition accuracy rates for the respiratory states using the four algorithms.**


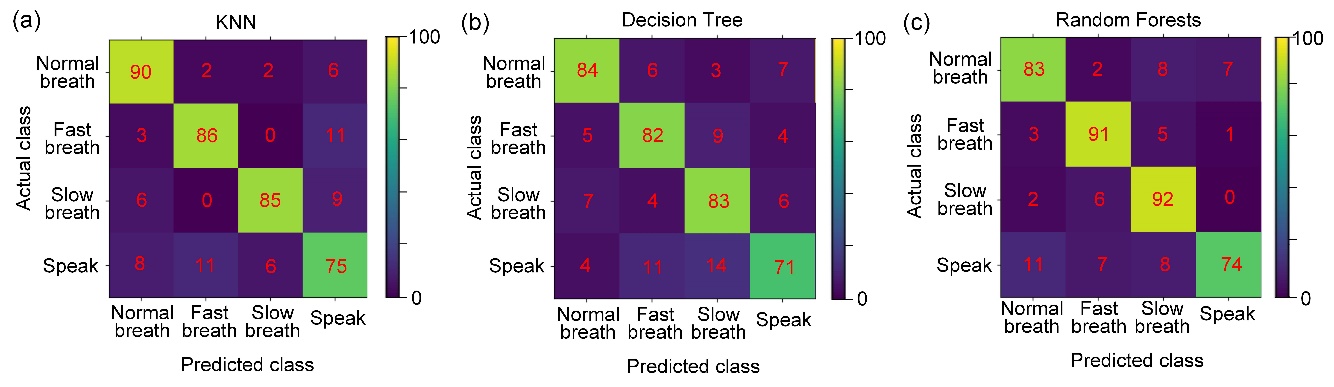


**Figure S12. Classification test confusion matrix with 400 groups of the dataset for recognition of four different respiratory states based on three different algorithms. The color bar represents the sizes of the predicted numbers.** (a) KNN algorithm. (b) Decision tree algorithm. (c) Random forests algorithm.

Tab. S1 Performance of humidity sensors based on different sensing materials

| **Material type** | **Sensing element** | **Sensitivity** | | **Recovery**  **Time (s)** | **Resolution**  **(%RH)** | | **Ref** |
| --- | --- | --- | --- | --- | --- | --- | --- |
| PI | capacitance | 0.01pF/%RH (10%-90%RH) | | 15 | - | | [1] |
| PI nanostructure | capacitance | 0.077pF/%RH (50%-85%RH) | | 7 | - | | [2] |
| P(VDF-TrFE) nanocone | capacitance | 0.0015pF/%RH (50%-90%RH) | | 3.430 | - | | [3] |
| AAO | capacitance | 3.3nF/%RH  (20%-60%RH) | 41.6nF/%RH  (60%-80%RH) | 11 | - | | [4] |
| ZnO NRs/WS_2_ | capacitance | 0.1pF/%RH  (50%-85%RH) | | 25.67 | - | | [5] |
| VS_2_ nanosheets | resistance | 0.24kOhm/%RH  (0%-50%RH) | 6.2kOhm /%RH  (50%-100%RH) | 12-50 | - | | [6] |
| Polymer/TiO_2_ | capacitance | 1.24pF/%RH (10%-90%RH) | | 25 | - | | [7] |
| Al_2_O_3_ | capacitance | 0.8pF/%RH  (10%-40%RH) | 27.5pF/%RH  (40%-90%RH) | 16.5 | - | | [8] |
| MoS_2_ | resonant frequency | 778Hz/%RH | | 8 | 0.3  (10%-30%RH) | 0.025  (70%-90%RH) | [9] |
| Carbon ink | resistance | - | | 14 | 1 | | [10] |
| Cellulose acetate butyrate | wave-  length shift | 0.307nm/%RH | | - | 0.06 | | [11] |
| Nanoforests | capacitance | 0.014pF/%RH  (10%-40%RH) | 0.11pF/%RH  (40%-90%RH) | 5 | 0.72  (10%-40%RH) | 0.075  (40%-90%RH) | This work |

**References:**

[1] Huang, J. Q., Chen, W. H., Zhu, D. P. & Han, L. A CMOS interdigital capacitive humidity sensor enhanced by a multi-layered structure. IEEE Sens., 1459-1462 (2014).

[2] Lee, H., Lee, S., Jung, S., Lee, J. Nano-grass polyimide-based humidity sensors. Sens. Actuat. B Chem. 154, 2-8 (2011).

[3] Niu, H. et al. Ultrafast-response/recovery capacitive humidity sensor based on arc-shaped hollow structure with nanocone arrays for human physiological signals monitoring. Sens. Actuat. B Chem. 334, 129637 (2021).

[4] Chung, C. K., Ku, C. A., Wu Z. E. A high-and-rapid-response capacitive humidity sensor of nanoporous anodic alumina by one-step anodizing commercial 1050 aluminum alloy and its enhancement mechanism. Sens. Actuat. B Chem. 343, 130156 (2021).

[5] Muhammad, A. D., Farah, F., Cuk I., Vivi F. The enhanced performance of capacitive-type humidity sensors based on ZnO nanorods/WS2 nanosheets heterostructure. Sens. Actuat. B Chem. 310, 127810 (2020).

[6] Feng, J. et al. Giant moisture responsiveness of VS2 ultrathin nanosheets for novel touchless positioning interface. Adv. Mater. 24(15), 1969-1974 (2012).

[7] Tian, Q. et al. High-performance porous MIM-type capacitive humidity sensor realized via inductive coupled plasma and reactive-ion etching. Sens. Actuat. B Chem. 258, 704-714 (2018).

[8] Kumar S., Raina K. K., Islam T. Anodic aluminium oxide based humidity sensor for online moisture monitoring of power transformer. Sens. Actuat. B Chem. 329, 128908 (2021).

[9] Li. D. et al. High resolution and fast response of humidity sensor based on AlN cantilever with two groups of segmented electrodes. IEEE Electron. Device. Lett. 99, 1-1(2021).

[10] Duan. Z. et al. Daily writing carbon ink: Novel application on humidity sensor with wide detection range, low detection limit and high detection resolution. Sens. Actuat. B Chem. 339, 129884 (2021).

[11] Wei, X., Huang, W. B., Huang, X. G., and Yu, C. Y. A simple fiber-optic humidity sensor based on extrinsic Fabry–Perot cavity constructed by cellulose acetate butyrate film. Opt. Fiber Technol. 19, 583-586 (2013).
